# Early clinical experience with varian halcyon V2 linear accelerator: Dual‐isocenter IMRT planning and delivery with portal dosimetry for gynecological cancer treatments

**DOI:** 10.1002/acm2.12747

**Published:** 2019-10-29

**Authors:** Hayeon Kim, M. Saiful Huq, Ron Lalonde, Christopher J. Houser, Sushil Beriwal, Dwight E. Heron

**Affiliations:** ^1^ Department of Radiation Oncology UPMC Hillman Cancer Center University of Pittsburgh School of Medicine Pittsburgh PA USA

**Keywords:** dual‐isocenter, extended‐field IMRT, gynecological cancer, Halcyon 2.0, *In vivo* portal dosimetry

## Abstract

**Purpose:**

Varian Halcyon linear accelerator version 2 (The Halcyon 2.0) was recently released with new upgraded features. The aim of this study was to report our clinical experience with Halcyon 2.0 for a dual‐isocenter intensity‐modulated radiation therapy (IMRT) planning and delivery for gynecological cancer patients and examine the feasibility of *in vivo* portal dosimetry.

**Methods:**

Twelve gynecological cancer patients were treated with extended‐field IMRT technique using two isocenters on Halcyon 2.0 to treat pelvis and pelvic/or para‐aortic nodes region. The prescription dose was 45 Gy in 25 fractions (fxs) with simultaneous integrated boost (SIB) dose of 55 or 57.5 Gy in 25 fxs to involved nodes. All treatment plans, pretreatment patient‐specific QA and treatment delivery records including daily *in vivo* portal dosimetry were retrospectively reviewed. For *in vivo* daily portal dosimetry analysis, each fraction was compared to the reference baseline (1st fraction) using gamma analysis criteria of 4 %/4 mm with 90% of total pixels in the portal image planar dose.

**Results:**

All 12 extended‐field IMRT plans met the planning criteria and delivered as planned (a total of 300 fractions). Conformity Index (CI) for the primary target was achieved with the range of 0.99–1.14. For organs at risks, most were well within the dose volume criteria. Treatment delivery time was from 5.0 to 6.5 min. Interfractional *in vivo* dose variation exceeded gamma analysis threshold for 8 fractions out of total 300 (2.7%). These eight fractions were found to have a relatively large difference in small bowel filling and SSD change at the isocenter compared to the baseline.

**Conclusion:**

Halcyon 2.0 is effective to create complex extended‐field IMRT plans using two isocenters with efficient delivery. Also Halcyon *in vivo* dosimetry is feasible for daily treatment monitoring for organ motion, internal or external anatomy, and body weight which could further lead to adaptive radiation therapy.

## 1 INTRODUCTION

The Halcyon 2.0 (Varian Medical Systems, Palo Alto, CA, USA) became clinically available in the market since mid‐July 2018 with new upgraded features over the previous version (1.0). Our institution installed Halcyon 2.0 in July 2018 as one of the first users. The upgraded features from Halcyon 1.0 to 2.0 are as follows: (a) kilovoltage (kV) imaging capability (Table 1), (b) maximum treatment length of 36 cm using multiple isocenters, (c) 0.5‐cm MLC effective resolution, and (d) dynamic beam flattening sequences that flatten the treatment field beam profiles for three‐dimensional (3D) conformal planning. Most other parts remain the same as 1.0 for hardware, beam data/modeling, MLC characteristics (dimension, dosimetric gap, transmission, interleaf leakage), integrated electronic portal imaging device (EPID) with portal dosimetry and treatment workflow.^1^, ^2^

**Table 1:** KV cone beam computed tomography characteristics on Halcyon V2.

Modes	11 clinical protocols
Energy	80–140 kVp
Scan time	From 16.6 s (Head, Breast, Thorax modes) to 40.6 s (Pelvis Large mode)
Scan range	24.5 cm
Scan diameter	49.1 cm
Imager	17.5‐cm lateral offset
Bow‐tie	Half bow tie/titanium filter
Pixel resolution	1280 × 1280 (43 cm × 43 cm panel)
Reconstruction	2‐mm slice thickness
Reconstruction algorithm	Conventional FDK (CBCT), aiterative process (iCBCT; nonlinear/statistical)

(2D KV orthogonal images are not available for clinical use).

^a^ New algorithm (iterative statistical reconstruction) added: Designed to remove noise and enhance image quality with high resolution.

Halcyon linear accelerator is mainly designed for intensity‐modulated radiation therapy/volumetric modulated arc therapy (IMRT/VMAT) delivery due to its unique features such as fast delivery via 4 RPM with a dose rate of 800 MU/s, FFF only beam, MLC characteristics, and automated daily IGRT workflow. The accuracy of IMRT/VMAT planning and delivery depends on the quality of treatment planning system commissioning based on the beam data acquisition and modeling.^3^, ^4^, ^5^, ^6^, ^7^ The Halcyon linac is preconfigured with a reference beam model built in the Eclipse treatment planning system which the users cannot modify. Thus, beam model parameters related to small fields and MLC dosimetry which typically are challenging for IMRT/VMAT commissioning can benefit in achieving good agreements between planning and delivery. Several studies have shown good agreements between measurements and calculated or reference values on Halcyon 1.0.^8^, ^9^

Halcyon 2.0 is capable of treating >28 cm treatment length using a dual isocenter (allowing maximum treatment length of 36 cm) which is limited to maximum field size of 28 × 28 cm^2^ on Halcyon 1.0. To accomplish this, large fields are split into a few smaller fields to treat larger than the maximum field size (i.e., 28 × 28 cm^2^) using two isocenters. For IMRT fields, the auto feathering technique has been introduced for splitting large fields. It involves splitting the beam into components with an overlap between them and with variable intensity in the overlap region. During an IMRT optimization, the dose objectives for dose gradients in the junction region are established, and dose is “feathered,” where a dose gradient is generated in abutting fields to obtain a uniform dose in the target so that the junction target dose can be controlled uniformly without hot or/and cold spots.^10^, ^11^, ^12^, ^13^ Eclipse planning system 15.6 utilizes autofeathering technique for dual‐isocenter IMRT planning with Halcyon 2.0.

Device‐based pretreatment patient‐specific quality assurance (QA) measurements have been widely practiced and is an accepted standard of care.^14^ Portal dosimetry using EPID has been also used for pretreatment patient‐specific QA.^15^, ^16^, ^17^ EPID‐based portal dosimetry checks need a separate portal dose image prediction algorithm to calculate portal doses using the fluence map for the field that requires additional calibration and commissioning. EPID‐based portal image dosimetry is also utilized for performing *in vivo* dosimetry using separately available commercial systems or in‐house developed software.^18^ Portal image dosimetry in Halcyon is one of the many unique features that is integrated with EPID and Eclipse planning system using AAA (Analytical Anisotropic Algorithm) dose calculation algorithm. EPID‐based portal dosimetry with Halcyon makes portal image dosimetry convenient, effective, and more efficient because it is performed by default unlike other conventional linac‐based EPID portal dosimetry. Therefore *in vivo* EPID‐based portal dosimetry on Halcyon can be used to assess daily treatment delivery efficiently.

Extended‐field IMRT is commonly used for gynecological cancer patients with pelvic and/or para‐aortic lymph node involvement. Traditionally, extended‐field radiotherapy has been delivered with anterior–posterior opposed fields including the para‐aortic lymph nodes. With this technique, generous portions of the small bowel have been included in the treatment field, causing significantly increased toxicities. The use of IMRT has shown better pelvic and para‐aortic region dose conformity while sparing critical organs like small bowel, kidneys, marrow, rectum, and bladder, resulting in decreased acute and late gastrointestinal morbidities.^19^, ^20^, ^21^ Extended‐field IMRT needs a dual isocenter with Halcyon due to its field size limit. As serving a high‐volume center for gynecological cancer treatments, we report our clinical experience with Halcyon 2.0 focusing on a dual‐isocenter IMRT planning and delivery for gynecological cancer patients and examine the feasibility of *in vivo* portal dosimetry. To the best of our knowledge, there are no reports available regarding Halcyon 2.0 clinical use experiences with the use of dual‐isocenter IMRT treatments.

## 2 MATERIALS AND METHODS

Twelve gynecological cancer patients were treated with extended‐field IMRT technique using two isocenters on Varian Halcyon 2.0. Treatment plans were generated with Eclipse v 15.6 planning system. All plans, treatment delivery records, and *in vivo* portal image dosimetry were retrospectively reviewed.

### 2.1 Patient characteristics and prescription

Three cervical and nine endometrium cancer patients were planned and treated with extended‐field IMRT. Treatment was planned for pelvis and pelvic/or para‐aortic node regions and the prescription dose was 45 Gy in 25 fractions (fxs) with a simultaneous integrated boost (SIB) dose of 55 or 57.5 Gy in 25 fxs to the involved nodes (Table 2). Planning CT scan was done with full bladder and empty rectum, and the same filling status was instructed for daily treatment as well.

**Table 2:** Treatment characteristics.

Patient	Prescription dose for PTV1 (SIB PTV2)	SIB targets (PTV2)	Number of beam angles (number of fields)	Distance between the two isocenters (cm)	Imaging mode and scan time^a^
1	45 (55) Gy	Pelvic and Para‐aortic nodes, other involved nodes	9 (18)	8	Pelvis iCBCT (36.7 sec)
2	45 (55) Gy	Pelvic and Para‐aortic nodes, other involved nodes	9 (18)	8	Pelvis iCBCT (36.7 sec)
3	45 Gy	Pelvic nodes (same dose as PTV1)	9 (18)	8	Pelvis iCBCT (36.7 sec)
4	45 (57.5) Gy	Pelvic and Para‐aortic nodes, other involved nodes	9 (18)	8	Pelvis iCBCT (36.7 sec)
5	45 (55) Gy	Pelvic and Para‐aortic nodes, other involved nodes	9 (18)	8	Pelvis iCBCT (36.7 sec)
6	45 (55) Gy	Pelvic and Para‐aortic nodes, other involved nodes	9 (18)	8	Pelvis iCBCT (36.7 sec)
7	45 (55) Gy	Pelvic and Para‐aortic nodes, other involved nodes	9 (18)	5.5	Pelvis iCBCT (36.7 sec)
8	45 (55) Gy	Pelvic and Para‐aortic nodes, other involved nodes	9 (18)	7	Pelvis iCBCT (36.7 sec)
9	45 (57.5) Gy	Pelvic and Para‐aortic nodes, other involved nodes	9 (18)	8	Pelvis iCBCT (36.7 sec)
10	45 Gy	Pelvic and Para‐aortic nodes (same dose as PTV1)	9 (18)	8	Pelvis iCBCT (36.7 sec)
11	45 (55) Gy	Pelvic and other involved nodes	9 (18)	5	Pelvis iCBCT (36.7 sec)
12	45 (55) Gy	Pelvic and Para‐aortic nodes, other involved nodes	9 (18)	8	Pelvis iCBCT (36.7 sec)

iCBCT: iterative CBCT; SIB: simultaneous integrated boost.

a 360‐full rotation for any mode.

### 2.2 Planning process

Following the institutional CT simulation procedure, the original (or the first) isocenter was placed at the time of CT scan by an attending physician. All patients were positioned in treatment position. For immobilization, patients had both arms up and held a wing board with a head rest. Feet were tied, and a knee cushion was used. We measured the distance between the inferior and superior target borders and placed the second isocenter to the desired position by shifting along the longitudinal direction (up to 8 cm separation allowed between the two isocenters with a treatment length of 36 cm) by verifying the dicom coordinates. Although the imaging isocenter can be placed at either of the two isocenters or in between the two isocenters, in the present study the imaging isocenter for all cases was placed at the same position as the first isocenter and daily CBCT imaging was performed at the imaging isocenter.

Only multibeam (fixed gantry) static IMRT technique was used for the present study cohort. For each patient, nine gantry angles per isocenter were set in equal distance with 40° apart, 180°, 140°, 100°, 60°, 20°, 340°, 300°, 260°, 220°, with a total of 18 beams for 2 isocenters (Table 2). Collimators were rotated between angles by 10°–20° (if necessary) to conform target shape better and spare OARs further. For the planning objectives, target doses were set for D95% (dose covering 95% of target volume) ≥ 95 % of their respective prescription doses (PTV1 with 45 Gy and SIB PTV2 with 55 or 57.5 Gy) and maximum dose ≤ 110 %. For organs at risk (OARs), bone marrow, small bowel, rectum, bladder, kidneys, femoral heads, and spinal cord were included for planning optimization process. Patient‐specific sets of optimization weights for the different objectives were used. Optimization weights were determined using the highest priorities for PTV objectives. OAR objectives were based on the optimization dose volume criteria based on the institution clinical pathway (Table 3). Treatment plans were generated using photon optimization algorithm (v. 15.603) with selecting autofeathering option during IMRT optimization and AAA dose calculation algorithm (v.15.603) was used for final dose calculation. For daily imaging, KV CBCT was scheduled to be used for all patients. It should be noted that iterative CBCT (iCBCT) technique has been newly added to Halcyon and its algorithm is designed to reduce noise in the image through iterative (statistical) reconstruction process and produce enhanced image quality with high resolution.

**Table 3:** Planning objectives for organs at risk (OARs).

Organ	Max dose (Gy)	Dose‐Volume limit (Gy)	Maximum volume at limit (%)
Bone marrow	55	20	75
Small Bowel	55	35	35
Rectum	47	40	60–65
Bladder	47	40	60–80
Kidney — left	45	16	5
Kidney — right	45	16	5
Femoral Head — left	40–45	25–35	5
Femoral Head — right	40–45	25–35	5
Spinal Cord	45	0	0

Dose constraints for the prescription dose of 45 Gy for PTV1 and SIB dose of 55 Gy for PTV2.

### 2.3 Pretreatment patient‐specific quality assurance (QA) measurements

Quality assurance plans were created for the MatriXX ion chamber array device using MultiCube phantom (IBA Dosimetry) and for portal dosimetry. The MatriXX has 1020 ion chamber arrays with 7.6 mm spacing in a 24 cm × 24 cm array with 0.08 cc volume per ion chamber. Both MatriXX‐based QA and portal image dosimetry using EPID were performed. Point doses were evaluated with 5% criteria, and for planar dose measurements gamma analysis was conducted using the criteria of 3%/3 mm and 3%/2 mm with 95% of total pixels in the plane for MatriXX‐based QA and portal dosimetry, respectively.

### 2.4 Megavoltage imager characteristics

The Halcyon digital megavoltage imager (DMI) has a 43 cm × 43 cm dimension mounted on a beam stopper. It has 1280 × 1280‐pixel resolution having 0.34 mm/pixel at the panel and 0.22 mm/pixel at isoplane. The imager has enhanced back scatter removal with 16‐bit images achieving a frame rate of up to 20 frames per second.

### 2.5 Review of daily treatment exit dose using *in vivo* portal image dosimetry

For each fraction, each treatment field portal image was reviewed, and all fields were combined to make a composite field. These were compared to the reference baseline (1st fraction) by the image planar dose using gamma analysis with the criteria of 4%/4 mm and 90% of total pixels for the composite field.

## 3 RESULTS

Extended‐field IMRT plans for all 12 patients met the planning criteria and were approved by the treating physician. A total of 300 fractions were delivered as planned.

### 3.1 Evaluation of treatment plans

Target dose metrics were as follows. D95% for PTV1 and SIB PTV2 had the ranges of 45.0–45.6 Gy (100–101.3%) and 55.1–58.3 Gy (100.2–101.3% of SIB prescription dose), respectively. Conformity Index (CI) for PTV1 was achieved with the range of 0.99–1.14. Homogeneity index (HI)^22^ for PTV1 was between 0.05 and 0.33 (HI = 0 as the perfect uniformity of dose in the target indicated by the squareness of the DVH defined by ICRU 83). The summary of plan quality for target doses and OARs is also presented in Tables 4 and 5. For OARs, most were well within the dose volume criteria. The largest deviation was the maximum point dose for rectum for one patient and it was 20% higher than the maximum planning goal due to its relatively large overlap with PTV1 (Table 4).

**Table 4:** Plan quality results for organs at risk (OARs).

Organ	Max dose plan/goal: range for all patients	Dose‐volume plan/goal: range for all patients	# Patients> 1.0	Maximum deviation (%)
Bone marrow	0.86–1.03	0.75–1.02	3	3.0
Small Bowel	0.84–1.07	0.15–0.8	2	7.0
Rectum	0.98–1.05	0.68–1.2	5	20.0
Bladder	0.99–1.11	0.59–1.13	6	13.0
Kidney — left	0.49–0.9	0–0.8	0	0
Kidney — right	0.4–0.95	0–0.94	0	0
Femoral Head — left	0.64–1.08	0–0.94	1	8.0
Femoral Head — right	0.69–1.08	0–0.98	1	8.0
Spinal Cord	0.70–1.0	0	0	0

Max dose plan/goal = ratio of plan results to the objectives (i.e. 1.03 = 3% higher maximum dose than planning objective (goal), 1.0 = same between the plan result and the goal).

Dose‐volume plan/goal = ratio of plan results to the objectives (i.e., 1.02 for Bone marrow means 2% higher maximum volume at dose volume limit than planning objective (goal). That is, 20 Gy at 75% is the goal, but achieved 20 Gy at 76.5% volume; 75.5/75.0 = 1.02).

**Table 5:** Plan quality results for targets (PTV1 and SIB PTV2).

Patient	D2 % (Gy)	D98 % (Gy)	D50 % (Gy)	HI	CI	D95 % (Gy)	SIB D95 % (Gy)	SIB max dose (Gy)
1	53	44.4	46.6	0.18	1.01	45	56	58.3
2	55.3	44.6	46.5	0.23	1.04	45.2	55.6	57.6
3^a^	47.4	44.4	46.3	0.06	0.99	45.1	n/a	n/a
4	60.5	45.2	47	0.33	1.14	45.6	57.8	59.9
5	47	44.5	46	0.05	1.02	45.1	55.1	57
6	49.5	44	46.2	0.12	1.01	45	55.6	58.7
7	53.5	45	46.3	0.18	1.07	45.5	55.3	57.6
8	56.5	44.8	46.7	0.25	1.03	45.2	55.7	58.5
9	60.1	44.6	47.1	0.33	1.01	45.1	58.3	61.8
10^a^	48.5	44.4	47.0	0.09	1.0	45.0	n/a	n/a
11	55.0	44.5	46.7	0.22	1.03	45.3	55.9	58.6
12	57.5	44.9	47.0	0.27	1.05	45.2	55.6	58.7
Mean (SD)	53.7 (4.7)	44.6 (0.32)	46.6 (0.36)	0.19 (0.1)	1.03 (0.04)	45.2 (0.19)	56.1 (1.07)	58.7 (1.36)

HI: Homogeneity Index defined as uniformity of dose in the target indicated by the “squareness of the DVH”. (defined by ICRU Report 83 (reference 22); HI = D2%–D98%/D50%. Zero (HI = 0) indicates that the absorbed dose distribution is perfectly homogeneous); CI: Conformity Index calculated as the ratio of volume enclosed by 95% isodose to the total target volume. (CI = 1.0 the perfect conformity); SD: standard deviation.

a SIB dose for PTV2 is same as PTV1.

### 3.2 Pretreatment patient‐specific QA results

Pretreatment QA results are given in Table 6. Gamma dose evaluation analysis for both MatriXX ion chamber array device and portal dosimetry yielded a mean value of 99.0 % (ranges: 97.3%–99.9 %) and 100% passing rates, respectively. An automatic shift between the two isocenters was verified using the MatriXX device simulating the planned shift.

**Table 6:** Results of pretreatment quality assurance (QA) and *in vivo* dosimetry.

Patient	Treatment delivery time and total MU	Pretreatment QA (aD; gamma/point dose)	Pretreatment QA (bPD; gamma/point dose)	*In vivo* PD cOut of tolerance (Number of fractions)
1	4 min 58 s, 1995	97.6%/2.1%	100%/0.6%	0/25
2	5 min 05 s, 2321	99.0%/1.3%	100%/0.8%	0/25
3	5 min 35 s, 2762	99.4%/2.0%	100%/0.4%	0/25
4	5 min 30 s, 2405	98.7%/1.5%	100%/3.4%	0/25
5	5 min 10 s, 2587	97.3%/2.5%	99.7%/1.0%	0/25
6	5 min 40 s, 2601	99.8%/2.5%	100%/0.2%	2/25
7	6 min 25 s, 3808	97.9%/1.1%	99.9%/0.8%	0/25
8	5 min 15 s, 2557	99.9%/1.1%	100%/1.4%	6/25
9	6 min 20 s, 3622	99.9%/0.1%	100%/2.2%	0/25
10	5 min 10 s, 2177	99.9%/0.2%	100%/1.1%	0/25
11	5 min 02 s, 2104	99.1%/1.7%	100%/0.7%	0/25
12	5 min 05 s, 2179	99.8%/0.8%	100%/1.0%	0/25
Mean (SD)	Time: 5 min 26 s (29.5 sec), MU: 2593 (573)	99.0%/1.4%	100%/1.1%	Total = 8 fractions: 2.7 % out of tolerance

Gamma statistics criteria for pretreatment QA: 3 %/3 mm with 95% pixels for MatriXX device and 3%/2 mm with 95% pixels for portal dosimetry.

SD: standard deviation.

a D: matrixx device.

b PD: portal dosimetry.

c Out of tolerance criteria for *in vivo* dosimetry during the treatment: 4%/4 mm with 90% of total pixels.

### 3.3 Treatment delivery and *in vivo* portal dosimetry results

Prior to each treatment fraction, 3D CBCT for setup verification was acquired. We used iterative CBCT (iCBCT) pelvis mode for all patients for each fraction and it took 36.7 s for a full 360° rotation (Table 2). Reconstruction time was an additional 15 s before treatment was initiated. We have used KV CBCT mode for daily setup verification for all patients in our clinical setting; MV imaging was not utilized for daily IGRT in our practice because KV CBCT imaging provides better soft tissue contrast compared to MV imaging. KV CBCT dose is not included in dose calculation with Halcyon 2.0, while the MV imaging dose was integrated with the treatment plan for final dose calculation and not adjustable during the course if treated with the same plan.

All 12 patients were treated with 2 isocenters using 18 treatment fields with 9 gantry angles for each isocenter (multibeam or fixed gantry IMRT technique). Treatment delivery time was between 5 and 6.5 min (depending on total MUs; total MU ranges were between 1995 and 3808) with a constant dose rate of 800 MU/min (Table 6). After the first isocenter was treated, treatment couch was shifted automatically to the second isocenter for beam delivery.

**Figure 1:**
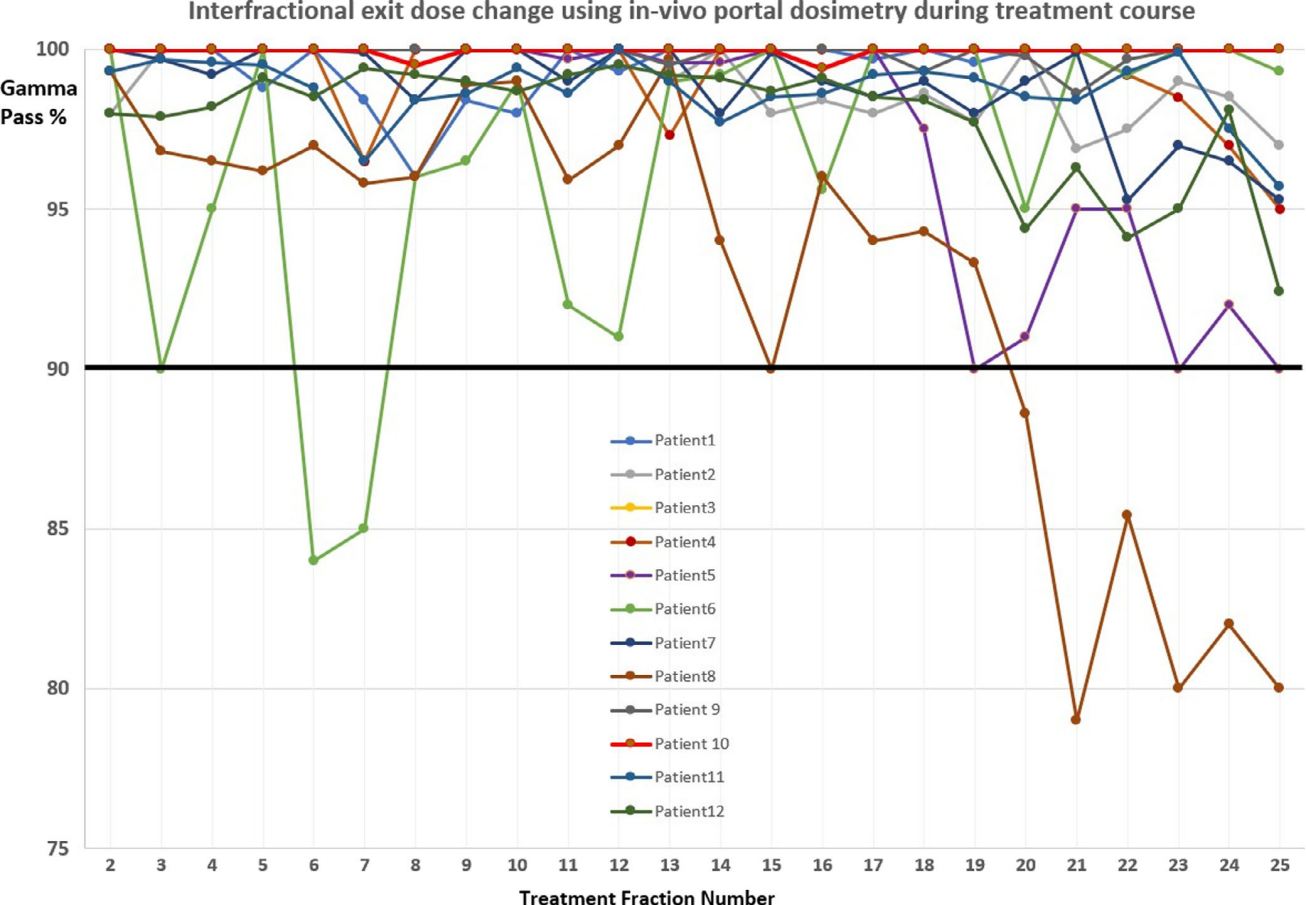
Interfractional exit dose change using *in vivo* dosimetry.

**Figure 2:**
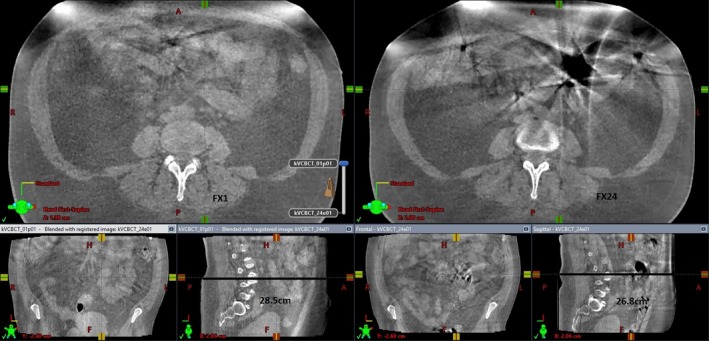
Comparison of cone beam computed tomography images for fraction 1 and fraction 24.

Eight out of total 300 fractions (2.7%) had a gamma passing < 90% of total pixels with 4 %/4 mm criteria (Table 6, Fig. 1). As can be seen in the Fig. 1, Interfractional *in vivo* dose variation was relatively larger for two patients (patient no. 6 with 2 fxs and patient no. 8 for 6 fxs) compared to other patients. Those fractions were found to have relatively larger small bowel filling change or motion compared to the baseline. In addition, SSDs were different by 1.5–2.0 cm as shown in Fig. 2 (line represented by the anterior and posterior direction at the isocenter) as an example. Additionally, we examined the *in vivo* dose variation with the pretreatment patient‐specific QA criteria using gamma passing rate of 3%/2 mm and 95% of total pixels in the plane. Thirty‐one fractions out of total 300 (10%) did not pass that criteria.

## 4 DISCUSSION

To the best of our knowledge, the present work is the first report on Halcyon V2 for a dual‐isocenter IMRT planning and delivery. Our results show that the dual‐isocenter technique on Halcyon V2 is not only effective to create complex extended‐field IMRT plans for gynecological cancers but it also facilitates a very efficient delivery. Faster beam delivery time can contribute to reduction in intrafraction motion of target and organs at risk, resulting in more accurate dose delivery. Furthermore, our study demonstrated that daily treatment delivery verification using *in vivo* portal dosimetry based on treatment exit dose is feasible, while most published studies reported the use of portal dosimetry only for pretreatment verification.^15^, ^16^, ^17^

**Figure 3:**
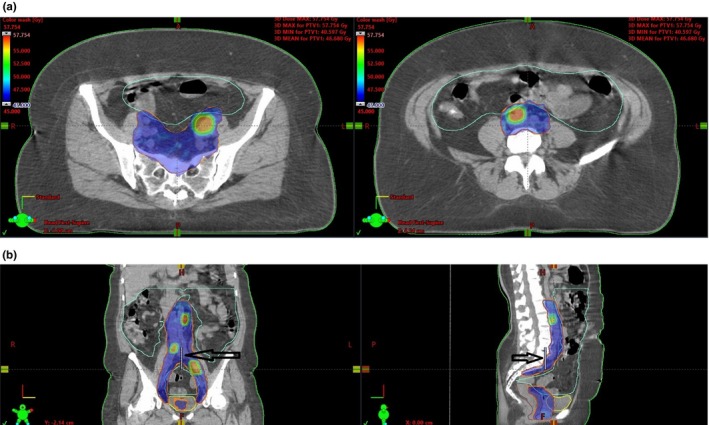
(a) Dose distribution at the two isocenters. (b). Dose distribution in the treatment field and junction area.

One of the limitations of the Halcyon linac is a relatively smaller field size (28 cm × 28 cm maximum) at the isocenter compared to conventional C‐arm linacs (40 cm × 40 cm). However, by utilizing a dual‐isocenter technique available with Halcyon 2.0, we were able to treat extended‐field IMRT cases for gynecological cancers; the maximum treatment length can be extended up to 36 cm with a maximum 8 cm difference between the two isocenters along the longitudinal direction. Junction area is autofeathered during the IMRT optimization. As seen from the plan quality review (Table 5), all 10 patients had a good CI between 0.99 and 1.14 (CI = 1.0 as the ideal reference; calculated as the ratio of volume enclosed by 95% isodose to the total target volume). Of note, HI had a higher variation with the ranges of 0.05–0.33 because 8 out of 10 cases had SIB targets covered with 122–128% (55 or 57.5 Gy; PTV2) of the primary target dose (45 Gy; PTV1). More importantly, use of the autofeathering technique during IMRT optimization has shown homogeneous and conformal doses to the targets without creating unnecessary hot or cold spots near normal tissues throughout the treatment field [Figs. 3(a) and 3(b)]. As seen in Figs. 3(a) and 3(b), PTV1 (orange colored contour) and PTV2 (SIB target with green colored contour) received 45 Gy represented by blue dose color wash and 55 Gy with red/yellow dose color wash, respectively. Particularly in Fig. 3(b), the line pointed by the arrow represents the distance between the two isocenters and that area along the line shows the junction area.

Pretreatment patient‐specific QA was performed with MatriXX ion chamber array device and portal dosimetry with EPID. For the dual‐isocenter technique, portal dosimetry using EPID cannot simulate a treatment couch shift but all treatment fields from two isocenters are delivered and combined at the same position of EPID. Thus, we performed a device‐based QA as well for dual‐ isocenter IMRT plans to verify the accurate couch position by shifting the MatriXX device simulating two isocenters and evaluating the dose distribution at the junction area. Due to the limit of the active measurement area on the Matrixx which exceeds the treatment field sizes, the main purpose of our pretreatment QA using the Matrixx for extended‐field IMRT was to ensure the dose distribution of junction area based on the planned couch shift between the two isocenters. The evaluation was conducted using the criteria of 3%/3 mm gamma analysis with 95% passing of total pixels and 5% for point dose difference, demonstrating all cases within tolerances (Table 6). Our pretreatment QA results are consistent with the recent report by De Roover et al.^8^ Their study with Halcyon 1.0 showed >96.5% and >92.5% for gamma analysis (3%/2 mm criteria) with portal dosimetry and device‐based patient‐specific QA for IMRT plans, respectively, and a point dose difference of <2.5% for the same plans.

AAPM TG 218 recommendations for IMRT QA methodologies and tolerance limits have been published recently.^14^ According to its recommendations, the true composite (TC) method, which consists of delivering all beams to a measurement device using the actual treatment beam geometry for the patient most closely simulates the treatment delivery to the patient. In addition, field‐by‐field (FF) analysis is useful to evaluate some subtle delivery errors for each treatment field which is highly modulated. EPID‐based portal dosimetry for patient‐specific QA is very convenient and efficient to measure the TC method and evaluate FF analyses compared to other device‐based measurement methods.

*In vivo* dosimetry using the integrated EPID showed a good consistency in daily treatment delivery overall. The interfractional *in vivo* exit dose change during the treatment course based on gamma analyses showed that 97.3% of total fractions (292 of 300 fractions) passed using the criteria of 4%/4 mm with 90% of total pixels in the dose plane (Table 6 and Fig. 1). The present study demonstrated that daily *in vivo* portal dosimetry based on exit dose is feasible with Halcyon 2.0. Halcyon EPID‐based *in vivo* dosimetry has the potential to monitor patients during complex treatments and possible (or significant) changes of organ motion, internal or external anatomy and body weight which could further lead to adaptive radiation therapy.

*In vivo* interfractional dose variation based on gamma analyses was found to be relatively higher for two patients only (Fig. 1). This suggests that these changes were not caused by systematic treatment delivery errors or dosimetric failures because the variation was found as a random pattern. Patient no. 8 had <90 % of total pixels passing with gamma analysis criteria in the dose plane for the last six fractions (fx 19–25) of the treatment course. We discovered that the SSD at the isocenter was changed by 1.5–2.0 cm for those fractions. This can be seen, for example, in Fig. 2 for patient no. 8 which shows a comparison of CBCT images between baseline (1st fraction; left side image) and the fx 24 (right side image); a SSD difference of 1.7 cm was measured on the lateral view images (thickness 28.5 cm for fx1 vs. 26.8 cm for fx24). In addition, small bowel motion and its filling status and shape were prominently different for fx 24 compared to the baseline. For those fractions which resulted in <90% of total pixels passing using the gamma analysis, small bowel motion and filling status were shown to have a relatively larger difference from a qualitative perspective compared to other fractions as seen in Fig. 2.

**Figure 4:**
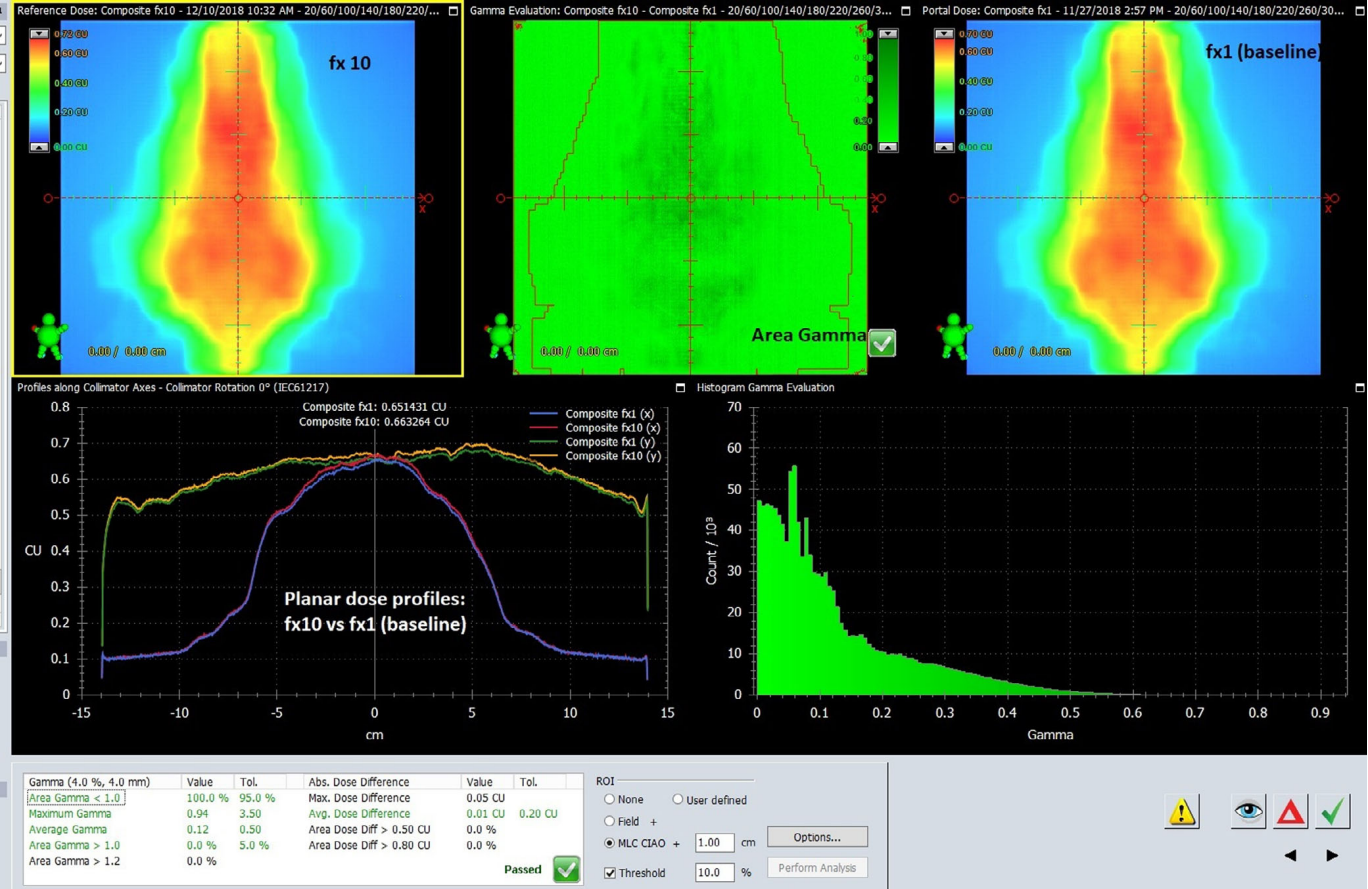
*In vivo* portal dosimetry comparison.

Figure 4 is an example of *in vivo* portal dosimetry demonstrating consistency in treatment delivery between fractions (patient no. 7). Upper left side image represents fraction 10 and right side fraction 1 (baseline). The center image was the result of gamma evaluation and 100% total pixels were passed (representing “area gamma < 1.0”) using gamma analysis of 4%/4 mm with 90% of total pixels criteria. The lower left image shows a planar dose profile comparison between the two fractions along the planes with x axis (left–right direction) and y axis (superior–inferior direction) area at the isocenters.

The recent study by Nailon et al.^18^ evaluated EPID‐based *in vivo* portal dosimetry for plans with various disease sites (n = 3795) using an independent commercial software (Dosimetry Check, Math Resolutions LLC, Columbia, MD) for portal dose calculations on Varian C‐series and Truebeam linear accelerators. Their results showed 96 % passing rate (4% failure; 153 out of total 3795 plans) between the planned dose calculated by the software and the delivered dose (*in vivo* dosimetry) on EPID. The threshold action level they set for plan evaluations was 10% for the percentage difference in point doses. Results of their analysis showed that breast patients had the highest discrepancy rate of 58% followed by head and neck cancer patients (18%). For female pelvis patients, they found that only seven patients (2%) exceeded the tolerance threshold; for prostate, 3% of the patients did not meet the tolerance criteria. Nailon et al.^18^ also found that interfractional dose deviations using *in vivo* dosimetry were mostly impacted by anatomic changes such as rectum and bladder filling or motion; this was not caused by procedural errors. In contrast to this study, analysis for the present study is based on gamma analyses criteria rather than analyzing only point dose difference; this provides better information for both quantitative and qualitative review for interfractional change as presented in Fig. 4. It should be noted that since extended‐field IMRT covers upper abdominal region, small bowel is the most critical organ with the possibility of larger variability for filling and motion in the present study.

All 12 patients were treated with two isocenters using 18 treatment fields via 9 gantry angles for each isocenter (multibeam or fixed gantry IMRT technique). Total beam delivery time recorded was between 5 and 6.5 min. iCBCT acquisition took 36.7 s (pelvis mode) with a full 360° rotation. For the present study, we used only multibeam IMRT (fixed gantry IMRT). This is because Varian technical advisory note indicated that there is a possibility of occurrence of erroneous hot spots in the peripheral region when rotational IMRT or VMAT techniques are used with Eclipse AAA dose calculation algorithm (versions 15.0–15.603). This problem has been addressed with a recent upgrade. This will facilitate the use of VMAT technique for planning and delivery which offers a faster delivery compared to multibeam/fixed gantry IMRT. Our experience with Halcyon VMAT delivery testing took less time with the less total number of MUs than multibeam IMRT plan and delivery. Thus, the treatment delivery time with two isocenters will further benefit using VMAT. Other studies published in the literature also reported reduced treatment time with Halcyon VMAT while maintaining comparable plan quality obtained with C‐arm linac‐based treatment.^23^, ^24^, ^25^

There are a few limitations in the present study. First, the *in vivo* portal dosimetry on the first fraction treatment was set as the reference for comparison with the consecutive fractions because it was not possible to calculate the predicted portal image dose from the planning system. If predicted portal dose could be calculated as a reference, the tolerance level criteria may be validated as in pretreatment patient‐specific QA. Therefore, calculated predicted portal dose would improve the quantitative evaluation of daily treatment verification with EPID on Halcyon. However, the present study was to assess interfractional *in vivo* dose variation and our approach using the first fraction as the baseline worked well for checking a consistency in the delivery during the treatment course. Second, the cohort of patients for the present study is relatively small, only 12 patients. Further investigation will be needed with a larger cohort for validation. In addition, we did not investigate the intrafractional motion during the treatment. However, several studies demonstrated that intrafractional motion did not cause substantial dosimetric change when treatment time was <10 min for pelvis treatments.^26^, ^27^, ^28^ We believe that intrafractional motion remains minimal for our study cohort due to relatively short treatment time. Next, we did not quantitate the correlation in detail between the *in vivo* portal dose and the anatomy change. The correlation between *in vivo* portal dose and the anatomy change could be quantitated; however, anatomy change can not only affect the daily *in vivo* portal dose but also setup uncertainty and other external factors may contribute to the interfractional dose variation. This subject needs a further research.

Lastly, the present study focused only on pelvic IMRT cases reflecting the high volume of gynecological cancer treatments in our clinic. Other disease sites may cause different results. However, based on the current study, two‐isocenter technique with Halcyon 2.0 was found to work well dosimetrically in this complex disease configuration and it is likely to work well for other disease sites.

## 5 CONCLUSION

Early clinical experience with Halcyon 2.0 linear accelerator was described in the present study, focusing on assessment of dual isocenter IMRT delivery for gynecological cancer patients. Halcyon 2.0 is not only effective to create complex extended‐field IMRT plans using dual isocenters but also performs an efficient and fast delivery. *In vivo* dosimetry integrated with EPID in Halcyon was evaluated and demonstrated feasibility for daily treatment monitoring. Some interfractional changes were detected in random pattern depending on internal organ motion (small bowel in this study) and SSD change. Halcyon EPID‐based *in vivo* dosimetry has the potential to function as an additional daily monitoring system for complex IMRT and adaptive radiotherapy. If predicted portal dose for treatment exit dose could be calculated, daily treatment *in vivo* verification will be accurately quantitated.

## CONFLICT OF INTEREST

The authors have no conflict of interest to declare.
